# Study of Symptomatic vs. Silent Brain Infarctions on MRI in Elderly Subjects

**DOI:** 10.3389/fneur.2021.615024

**Published:** 2021-02-17

**Authors:** Sheelakumari Raghavan, Jonathan Graff-Radford, Eugene Scharf, Scott A. Przybelski, Timothy G. Lesnick, Brian Gregg, Christopher G. Schwarz, Jeffrey L. Gunter, Samantha M. Zuk, Alejandro Rabinstein, Michelle M. Mielke, Ronald C. Petersen, David S. Knopman, Kejal Kantarci, Clifford R. Jack, Prashanthi Vemuri

**Affiliations:** ^1^Departments of Radiology, Mayo Clinic, Rochester, MN, United States; ^2^Neurology, Mayo Clinic, Rochester, MN, United States; ^3^Health Sciences Research, Mayo Clinic, Rochester, MN, United States; ^4^Information Technology, Mayo Clinic, Rochester, MN, United States

**Keywords:** silent brain infarction, clinical stroke, vascular distribution, middle cerebral artery, laterality

## Abstract

Brain infarctions are closely associated with future risk of stroke and dementia. Our goal was to report (i) frequency and characteristics that differentiate symptomatic vs. silent brain infarctions (SBI) on MRI and (ii) frequency and location by vascular distribution (location of stroke by major vascular territories) in a population based sample. From Mayo Clinic Study of Aging, 347 participants (≥50 years) with infarcts detected on their first MRI were included. Infarct information was identified visually on a FLAIR MRI image and a vascular territory atlas was registered to the FLAIR image data in order to identify the arterial territory of infarction. We identified the subset with a clinical history of stroke based on medical chart review and used a logistic regression to evaluate the risk factors associated with greater probability of a symptomatic stroke vs. SBI. We found that 14% of all individuals with infarctions had a history of symptomatic stroke (Silent: *n* = 300, symptomatic: *n* = 47). Factors associated with a symptomatic vs. SBI were size which had an odds ratio of 3.07 (*p* < 0.001), greater frequency of hypertension (odds ratio of 4.12, *p* = 0.025) and alcohol history (odds ratio of 4.58, *p* = 0.012). The frequency of infarcts was greater in right hemisphere compared to the left for SBI. This was primarily driven by middle cerebral artery (MCA) infarcts (right = 60%, left = 40%, *p* = 0.005). While left hemisphere strokes are more common for symptomatic carotid disease and in clinical trials, right hemispheric infarcts may be more frequent in the SBI group.

## Introduction

Brain infarcts are a common cerebrovascular pathology of aging and a common cause of cognitive impairment (1). The term “Silent brain infarcts” (SBI) (2–5), has been used to refer to infarcts which are observed on conventional MRI or CT but without known clinical symptoms. SBI make up the majority of infarcts in population-based studies (6–8) compared to clinically recognized infarcts. SBI have become an established risk factor for future symptomatic infarcts and cognitive decline. Several studies have highlighted the prevalence of infarcts detected in the general population (7, 9, 10). While stroke trials have revealed a greater likelihood of left cerebral hemispheric (LH) infarctions compared to right hemispheric (RH) infarctions (11, 12), the topography of SBI has been understudied. Our objective was to report on the frequency and characteristics that differentiate symptomatic vs. SBI on MRI and also explore their frequency and location by major vascular territories (vascular distribution). We hypothesized that the SBI would be overrepresented in the right hemisphere compared to the left hemisphere because individuals with right hemisphere strokes may be less likely to recognize non-dominant deficits.

## Methods

### Study Participants

All participant data were selected from the Mayo Clinic Study of Aging (MCSA), a population-based study of residents living in Olmsted County, Minnesota. The Rochester Epidemiology Project (REP) medical records linkage system was used to enumerate the MCSA population (13, 14), which followed an age and sex-stratified design. The REP allowed us to ascertain the history of vascular risk factors and the details have been published previously (15, 16). In MCSA, 1,845 elderly individuals (aged ≥ 50 years) had an infarction assessment. We selected 347 subjects and considered their first scan (FLAIR-MRI and T1-weighted MPRAGE imaging sequences) in which they had an infarction for this study. During the corresponding clinical visit 275 were cognitively unimpaired, 56 were diagnosed with mild cognitive impairment, and 16 had dementia. The diagnosis of the participants was based on the detailed clinical evaluation by the neurologist, assessment of neuropsychological test battery by the neuropsychologist, and a clinical dementia rating assessment by the study coordinator. The detailed diagnostic criteria for patients have been previously described (17).

### Potential Risk Factors

We determined the cardiovascular risk factors including hypertension, dyslipidemia, and diabetes mellitus using nurses that abstracted the REP medical-records linkage system as previously reported (18). Among these, 90% of hypertensive individuals were treated for hypertension. The ICD-9 and ICD-10 codes were searched also to determine the report of symptomatic strokes. Alcoholism was determined based on the combination of CAGE and the NHIS form. We also assessed smoking status based on self-reports of never smoking vs. former or currently smoking (19).

### Assessment of Infarcts on FLAIR MRI

All MRI images were acquired on 3T GE scanner (GE Medical Systems, Milwaukee, WI). The 2D T2-weighted FLAIR- MRI scans were obtained with the following parameters: repetition time = 11 000 ms, echo time = 147 ms, inversion time = 2,250 ms, 256 × 192 matrix, 24-cm field of view, and voxel size = 0.86 × 0.86 × 3 mm. The full details of infarct grading have been recently published (20). In brief infarcts were graded on two-dimensional FLAIR MRI that was co-registered with an MPRAGE (magnetization-prepared rapid gradient-echo) T1 MRI. All possible infarcts were initially identified by trained image analysts and subsequently confirmed by a vascular neurologist (JGR) to whom all clinical information was masked. The intra-rater reliability based on blinded reading of 50 possible infarcts on two separate occasions was excellent (κ statistic, 0.92).

Cortical infarctions were characterized as hyperintense T2 FLAIR lesions (gliosis) involving cortical gray matter that extended to the cortical edge with or without involvement of the underlying white matter. These infarctions were identified on the T2 FLAIR sequence, with a corresponding T1 hypointensity required for confirmation. The size of the cortical infarction was determined by measuring the largest diameter (in mm) of the hyperintensity/gliosis on the axial slice by considering the size on all the slices.

Subcortical infarctions were characterized as hyperintense T2 FLAIR lesions with a dark center, seen in the white matter, infratentorial, and central gray-capsular regions. The dark area (tissue loss) must be ≥3 mm in diameter as measured on the T2 FLAIR or T1, whichever image shows the findings more clearly. Subcortical infarcts were distinguished from perivascular spaces by size, location, and shape. The size of the subcortical infarction was determined by measuring the largest diameter (in mm) of the hypointensity/tissue loss on the axial slice by considering the size on all the slices.

### Assessment of Vascular Territory and Laterality on FLAIR MRI

We nonlinearly registered a vascular territory atlas (developed in-house, traced in MCALT space) (21) using a textbook reference (22) onto participants' T1-weighted image and transformed it onto the rigid-registered FLAIR image using ANTs (23). The atlas is divided into 14 regions including bilateral terminal and penetrating anterior cerebral artery (ACA), middle cerebral artery (MCA), anterior choroidal artery (AChA), posterior cerebral artery (PCA) and unknown (cerebellum, pons and medulla). We used this method for identifying the anatomical landmarks and then assigning the cortical and subcortical infarctions on the FLAIR image to the specific vascular territory. This technique assigned the anatomical landmarks into atlas space to distinguish the left and right ACA, MCA and PCA. The posterior fossa region infarctions were excluded in the vascular territory atlas. We further validated these vascular territory assessments with visual inspection by SKR.

### Statistical Analysis

Statistical analyses were performed with SAS and R. The continuous variables were summarized as mean and standard deviation and categorical variables as frequency and percentage. The variables were assessed for normality and log transformed for non-normal distribution. These were analyzed with either a two-sample two-sided *t*-test or chi-squared test. Next, we ran logistic regression model that included age, gender, education, and all of the vascular risk variables as predictors. Then, a step-wise elimination was done to form a parsimonious model with the significant predictors of clinical vs. silent stroke. This model was cross-checked with both forward and backward elimination that yielded the same final model, and odds ratios, associated 95% confidence intervals, and *p*-values were reported. The left and right distributions of cerebral infarcts were compared using McNemar's test for paired nominal data.

#### Standard Protocol Approvals, Registrations, and Patient Consents

The study was approved by the institutional review boards of Mayo Clinic and Olmsted Medical Center. Written informed consent was obtained from all participants/caregivers prior to taking part in the study.

#### Data Availability

Data from the MCSA, including data used in this study are available upon reasonable request via https://www.mayo.edu/research/centers-programs/alzheimers-disease-research-center/research-activities/mayo-clinic-study-aging/for-researchers/data-sharing-resources.

## Results

The demographic and clinical characteristics of the study population are shown in Table 1. Fourteen percent of all individuals with stroke on FLAIR MRI had clinically recorded stroke as ascertained by health care records or self-report. The mean age at the time of imaging, sex, and education were not significantly different between symptomatic and silent groups.

**Table 1:** Characteristics table of infarction subjects with the mean (SD) listed for the continuous variables and count (%) for the categorical variables.

	**Silent *n* = 300**	**Symptomatic *n* = 47**	**Total *n* = 347**	*P-value
Age, yrs	77.6 (8.7)	79.8 (7.9)	77.9 (8.6)	0.093
Males, no. (%)	188 (63%)	27 (57%)	215 (62%)	0.49
Education, years	14.5 (3.0)	14.3 (2.3)	14.5 (2.9)	0.57
Maximum diameter, mm	7.6 (6.7)	17.5 (14.3)	8.9 (8.8)	<0.001
Hypertension, no. (%)	227 (76%)	44 (94%)	271 (78%)	0.006
Diabetes Mellitus, no. (%)	56 (19%)	13 (28%)	69 (20%)	0.15
Atrial Fibrillation, no. (%)	51 (17%)	12 (26%)	63 (18%)	0.16
Dyslipidemia, no. (%)	245 (82%)	42 (89%)	287 (83%)	0.19
Coronary artery disease, no. (%)	107 (36%)	24 (51%)	131 (38%)	0.043
Smoking status, no. (%)	132 (44%)	27 (57%)	159 (46%)	0.085
Alcoholism, no. (%)	10 (3%)	8 (17%)	18 (5%)	<0.001
Antiplatelet, no. (%)	184 (61%)	35 (74%)	219 (63%)	0.083
Anticoagulants, no. (%)	25 (8%)	9 (19%)	34 (10%)	0.020
Any antithrombotic, no. (%)	197 (66%)	41 (87%)	238 (69%)	0.003
Both antithrombotic, no. (%)	12 (4%)	3 (6%)	15 (4%)	0.46

* *P-values for differences between groups are from a t-test for the continuous variables and a chi-squared test for the categorical variables*.

### Differentiating Characteristics Between Symptomatic and Silent Infarcts

Dichotomized analysis of common stroke risk factors, diabetes mellitus, atrial fibrillation, and smoking status were not significantly different between silent and clinical strokes. In group comparisons in Table 1, maximum infarct diameter (*p* < 0.001), hypertension (*p* = 0.006), alcoholism (*p* < 0.001), coronary artery disease (*p* = 0.04), anticoagulant (*p* = 0.02), and antithrombotic (*p* = 0.003) status were significantly different between the groups. When we ran a multivariate logistic regression model with all risk factors, we found only size of the infarction (OR = 3.07, *p* < 0.001), hypertension (OR = 4.12, *p* = 0.025), and alcoholism (OR = 4.58, *p* = 0.012) significantly predicted symptomatic stroke vs. SBI (Table 2).

**Table 2:** Logistic regression model with clinical stroke as outcome variable.

	**Odds ratio (95% CI)**	***P*-value**
Hypertension	4.12 (1.20, 14.21)	0.025
Alcoholism	4.58 (1.40, 15.01)	0.012
Maximum diameter	3.07 (1.97, 4.77)	<0.001

*Due to skewness maximum diameter was analyzed on the log scale*.

### Hemispheric Differences in the Vascular Distribution

**Figure 1:**
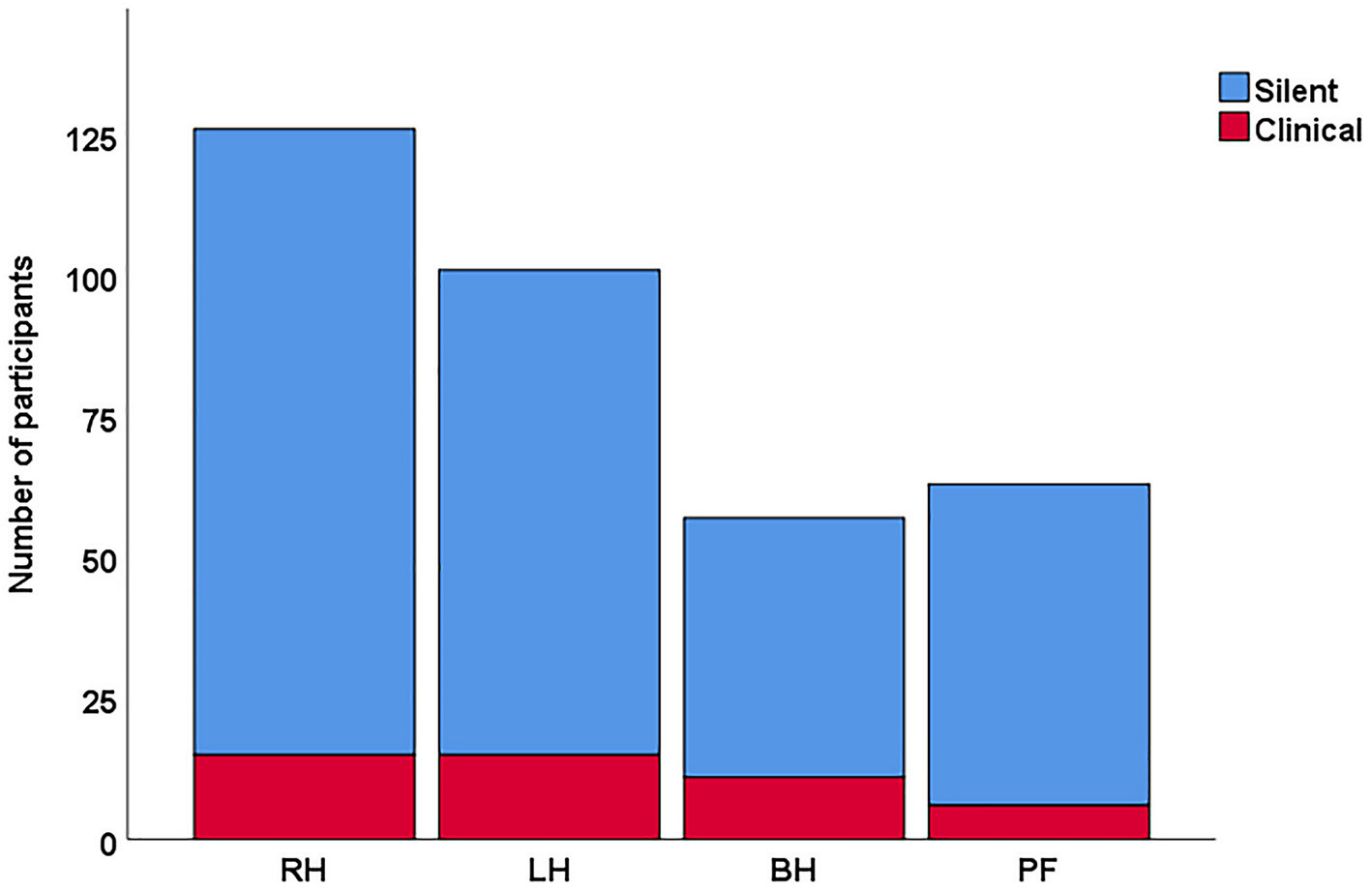
Distribution of overall stroke in silent vs clinical stroke participants. In this cohort, the overall frequency was higher for silent strokes especially in the right hemisphere. RH, right hemisphere; LH, left hemisphere; BH, both hemisphere; PF, posterior fossa.

Among the 347 participants, 36.3% (*n* = 126) had stroke only in the right hemisphere, 29.1% (*n* = 101) in only the left hemisphere, 16.4% (*n* = 57) in both hemispheres, and 18.2% (*n* = 63) in the posterior fossa region (cerebellum, brainstem, and pons) Figure 1. The bilateral and posterior fossa strokes were excluded from the subsequent analysis of hemispheric differences. Single hemisphere Infarcts were more common in the right MCA territory than left MCA territory [(right: 59.6% (*n* = 93), left: 40.4% (*n* = 63), *p* = 0.02] Table 3. Overall, right hemispheric infarction was observed more commonly in the silent brain infarct group [(right: 55.9% (*n* = 109), left: 44.1% (*n* = 86), *p* = 0.1], when the participants were divided into the silent and symptomatic stroke Table 4 (symptomatic findings are not shown). There were more MCA distribution was observed in RH (60%) than LH (40%), *p* = 0.005. However, the overall laterality was not different in the symptomatic stroke group with [right: 51.7% (*n* = 15), left: 48.3% (*n* = 14), *p* = 0.85]. The *p*-values for the symptomatic group need to be interpreted with caution due to low sample size.

**Table 3:** Hemispheric differences of vascular distribution in overall infarcts participants.

	**RH**	**LH**	**Total**	***P***	**Odds ratio**
Total	126 (55.5)	101 (44.5)	227	0.097	1.25
ACA	17 (48.6)	18 (51.4)	35	0.87	0.94
MCA	93 (59.6)	63 (40.4)	156	0.016	1.47
PCA	26 (47.3)	29 (52.7)	55	0.69	0.90

*Data values are presented as either n or n(%). ACA, anterior cerebral artery; MCA, middle cerebral artery; PCA, posterior cerebral artery. McNemar's test for paired nominal data*.

**Table 4:** Hemispheric differences of vascular distribution in silent stroke.

	**RH**	**LH**	**Total**	***P***	**Odd's ratio**
Total	109 (55.9)	86 (44.1)	195	0.10	1.27
ACA	15 (45.5)	18 (54.5)	33	0.60	0.83
MCA	81 (60)	54 (40)	135	0.005	1.5
PCA	18 (45)	22 (55)	40	0.53	0.82

*Data values are presented as either n or n(%). ACA, anterior cerebral artery; MCA, middle cerebral artery; PCA, posterior cerebral artery. McNemar's test for paired nominal data*.

## Discussion

The main findings of the present study were: (1) 14% of all individuals with infarction on FLAIR MRI in a population-based sample had clinically recorded strokes as ascertained by health care records. (2) After accounting for the greater size of symptomatic infarcts in comparison to SBI, hypertension and alcoholism also predicted the greater likelihood of a symptomatic vs. silent stroke. (3) Right-sided hemispheric infarctions were more common among SBI which was driven by the higher frequency in the MCA territory.

### Risk Factors for Symptomatic vs. Silent Strokes

In the present study, larger size of the infarct was a significant predictor of symptomatic stroke vs. SBI as expected. After controlling for the diameter of the infarct, hypertension and alcoholism were associated with symptomatic stroke. The association with hypertension corroborates previous risk factor profile studies on silent and symptomatic infarcts (10, 24, 25). Our study along with others (26, 27) detected alcoholism as a strong independent risk factor for stroke, both ischemic and hemorrhagic. The possible reason for this is that heavy drinking and chronic alcoholism is associated with other risk factors that lead to infarcts such as increase in blood pressure, frequency of atrial fibrillation, as well as sleep apnea and cardiomyopathy. Since our findings were limited by the lack of dose-response relationship, a detailed study with stroke subtypes may shed light on the possible mechanisms as suggested by the meta-analysis (28).

### Laterality of the Silent Strokes

Accumulating evidence on the hemispheric difference and stroke outcome (29–35) have shown that left hemisphere strokes are more commonly detected than right hemisphere strokes in clinical trials and hospital based cohorts. This apparent over-representation may be because of greater recognition of clinical deficits from the function of the dominant hemisphere (36). In contrast, anosagnosia is more common with right hemisphere infarcts (37), particularly those involving the MCA territory (38). Consistent with this hypothesis, we identified a greater proportion of right hemispheric strokes in a population-based study from MCSA with predominantly silent brain infarctions. This overrepresentation of non-dominant SBI has been observed in 848 subjects with asymptomatic high grade carotid stenosis (39).

The asymmetric pattern in our study was driven by the greater overall MCA territory distribution in SBI compared to ACA and PCA. It is well-known that MCA is the largest and most prevalent cerebral artery linked with infarcts (40–42). In contrast with our findings, a similar frequency in the left and right MCA territories were reported in the clinically silent TOAST study participants (43), although they used CT rather than MRI to define infarction and the TOAST study was not population-based. The findings from the Rotterdam study showed that the atherosclerotic plaque prevalence and thickness was greater in left than right (44). This may be due to a greater composition of calcification in the right-sided plaques, which are more stable and less vulnerable to cerebrovascular complications (45, 46). Previous studies have suggested that variability of carotid bifurcation anatomy might affect the development of atheroma (47) and ICA stenosis (48) and possibly affect the preferential laterality of the stroke. In patients acutely presenting to the hospital Hedna et al. (35) demonstrated greater frequency of left MCA strokes compared to right MCA strokes and the left MCA strokes presented with a higher NIHSS.

Notably, the asymmetric pattern in SBI patients has been evaluated with cortical thinning (49). By using the large deformation diffeomorphic metric mapping (LDDMM), Thong et al. (49) identified more widespread and severe RH atrophy patterns than LH in silent lacunar infarctions. However, the patterns were independent of the number of infarctions. Speculatively, the RH dominance either may be due to the age-related damage (50) or may be the greater impact of associated vascular pathologies in SBI (51). This may be part of our future research.

### Limitations

The present study has some limitations. Though we had a large sample of elderly individuals recruited from the population, the number of participants with symptomatic stroke in the subsample was smaller and was not sufficient to determine laterality differences in those with symptomatic stroke. We accounted for the size of the infarction by using the largest diameter on a slice because it was less time intensive to measure but other methods (35) may provide greater accuracy. Another limitation is that nonparticipation in MCSA could conceivably have included a greater proportion of individuals with overt infarcts. Had they been included our proportion of silent infarcts relative to overt ones might have been a bit lower.

## Conclusion

We report on the frequency, location, and characteristics of clinical vs. silent brain infarctions in a population based sample. The size and laterality between SBI and clinical infarcts differs in the general population. We found evidence for our hypothesis that right hemispheric infarcts may be more frequent in the SBI group which is in contrast to the left hemisphere strokes that are commonly seen with symptomatic carotid disease and in clinical trials.

## Data Availability Statement

The raw data supporting the conclusions of this article will be made available by the authors, without undue reservation.

## Ethics Statement

The studies involving human participants were reviewed and approved by Institutional review board, Mayo Clinic, Rochester. The patients/participants provided their written informed consent to participate in this study.

## Author Contributions

SR, JG-R, and PV conceived and designed the study. SR, JG-R, ES, SP, and TL drafted the manuscript and figures. All authors participated in data collection and analysis.

## Conflict of Interest

DK reported serving on a data safety monitoring board for the DIAN study, serving on a Data Safety monitoring Board for a tau therapeutic for Biogen, but receives no personal compensation, and serving as an investigator in a clinical trials sponsored by Lilly Pharmaceuticals and the University of Southern California, and receiving research support from the National Institutes of Health (NIH) outside the submitted work. JG-R reported receiving research support from the National Institute on Aging outside the submitted work. MM reported receiving research support from the NIH, Department of Defense, and unrestricted research grants from Biogen outside the submitted work. CJ reported serving on an independent data monitoring board for Roche, serving as consultant for Biogen, for Eli Lilly, and serving as a consultant and speaker for Eisai but receives no personal compensation from any commercial entity; he also reported receiving research support from the NIH and the Alexander Family Alzheimer's Disease Research Professorship of the Mayo Clinic. RP reported receiving consulting fees from Hoffman-La Roche Inc., Merck Inc., Genentech Inc., Biogen Inc., GE Healthcare, and Eisai Inc., outside the submitted work. PV reported receiving grants from the NIH during the conduct of the study. CS reported receiving funding from the NIH, unrelated to this study. The remaining authors declare that the research was conducted in the absence of any commercial or financial relationships that could be construed as a potential conflict of interest.
